# A Small-Divergence-Angle Orbital Angular Momentum Metasurface Antenna

**DOI:** 10.34133/2019/9686213

**Published:** 2019-11-15

**Authors:** Jianchun Xu, Ke Bi, Ru Zhang, Yanan Hao, Chuwen Lan, Klaus D. McDonald-Maier, Xiaojun Zhai, Zidong Zhang, Shanguo Huang

**Affiliations:** ^1^State Key Laboratory of Information Photonics and Optical Communications, School of Science, Beijing University of Posts and Telecommunications, Beijing 100876, China; ^2^Beijing University of Posts and Telecommunications Research Institute, Shenzhen 518057, China; ^3^Beijing Key Laboratory of Space-Ground Interconnection and Convergence, Beijing University of Posts and Telecommunications, Beijing 100876, China; ^4^School of Computer Science and Electronic Engineering, University of Essex, Colchester CO4 3SQ, UK; ^5^Key Laboratory for Liquid-Solid Structural Evolution and Processing of Materials (Ministry of Education), Shandong University, Jinan 250061, China

## Abstract

Electromagnetic waves carrying an orbital angular momentum (OAM) are of great interest. However, most OAM antennas present disadvantages such as a complicated structure, low efficiency, and large divergence angle, which prevents their practical applications. So far, there are few papers and research focuses on the problem of the divergence angle. Herein, a metasurface antenna is proposed to obtain the OAM beams with a small divergence angle. The circular arrangement and phase gradient were used to simplify the structure of the metasurface and obtain the small divergence angle, respectively. The proposed metasurface antenna presents a high transmission coefficient and effectively decreases the divergence angle of the OAM beam. All the theoretical analyses and derivation calculations were validated by both simulations and experiments. This compact structure paves the way to generate OAM beams with a small divergence angle.

## 1. Introduction

Light beams carrying orbital angular momentum (OAM) have attracted extensive attention owing to their special electromagnetic properties [1–4]. Since the first OAM beam was successfully simulated in the microwave band, there has been a growing interest in the study of radio OAM beams [5–7]. Due to the orthogonality between different radio OAM modes, every OAM beam with different modes can be regarded as a communication channel. Thus, infinite modes can provide infinite channels, which can infinitely expand the communication capacity without increasing the bandwidth [8, 9]. This characteristic of OAM beams may bring great advances in traditional wireless communication [10]. Although there are debates concerning the capability of OAMs to effectively increase the communication capacity of multiple-input-multiple-output wireless systems [11, 12], OAM beams also exhibit distinct advantages in many fields such as particle trapping, optical imaging, and microscopic particle rotation [13–16].

To date, various methods have been created to generate OAM beams such as spiral phase plates, spiral reflectors, computer-generated holograms, antenna arrays, and metasurfaces [17–20]. Among them, the spiral phase plates and spiral reflectors are bulky and difficult to process. The important losses caused by transmission or reflection prevent practical applications. The computer-generated holograms and optical mode conversion are applicable in the optical frequency range. Compared to other methods, the antenna array generally requires a complex feed network [21].

A metasurface is a kind of periodic or quasi-periodic structure that can achieve special properties that do not exist in natural materials [22, 23]. Its planar structure presents a low profile, and small unit cells can accurately modulate the phase and amplitude of the electromagnetic (EM) wave [24–26]. Thus, metasurfaces have been widely applied in polarization converters, flat lenses, and OAM beam generators [27–30]. Moreover, the OAM beams generated by metasurfaces show great performance in terms of pure, transmissivity, reflectivity, and shape of the phase front [31, 32]. Multimode OAM beams can be generated by one single metasurface [33–36]. However, metasurfaces usually require at least hundreds of unit cells to obtain an accurate modification, which causes great challenges in terms of design and manufacture. In addition, the divergence angle of the OAM beam needs to be improved for its practical applications.

Herein, a metasurface antenna is proposed to simplify the conventional metasurface and decrease the divergence angle of a generated OAM beam. In the structure of the unit cell, multilayer ring gaps are used to obtain high transmittance and enough phase variation for the metasurface design and OAM beam generation. The phase difference distributed in the circle can engender the OAM beam generation, and the phase gradient in the radial direction is beneficial to decrease the divergence angle. Here, the phase gradient is innovatively applied in the OAM generation. The reasonable arrangement can simultaneously realize the OAM generation and the small divergence angle. This divergence angle adjustment of the OAM beam is carried out by the transmitted phase gradient, which distinguishes our work from other OAM metasurface designs. The theoretical calculations, simulations, and experiments of the proposed metasurface antennas are performed to demonstrate the proposed principle. This proposed method provides a simplified method to design OAM metasurface antennas with a small divergence angle.

## 2. Materials and Methods

**Figure 1:**
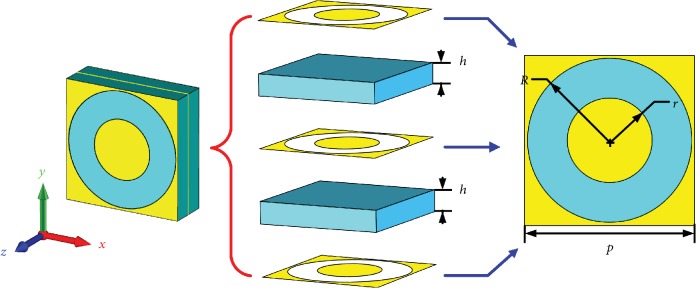
Structure of the circular slot element.

The circular slot structure is compact and suitable for steering EM waves with high efficiency [37]. It can manipulate EM waves on the subwavelength scale. Moreover, the multilayer structure is favorable to achieve broadband transparency and provides enough phase variation due to the coupling among the cascaded layers, which is essential for the OAM metasurface antenna design. Thus, this multilayer circular slot structure is used for the unit cells of the metasurface.  Figure 1 illustrates the configuration of the circular slot element. This unit cell is composed of three metallic layers with a thickness of 0.018 mm and two dielectric layers with a thickness of *h* = 2 mm. Each layer has a side length of *p* = 12 mm, and the dielectric layers have a permittivity of 2.65.

**Figure 2:**
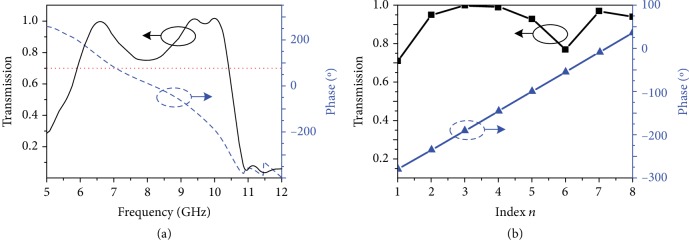
(a) Simulated transmission amplitude (black solid line) and phase (blue dashed line) for *r* = 5.8 mm. (b) Simulated transmission amplitude (black squares) and phase difference (blue triangles) of the unit cell with various *r*_*n*_ at 10 GHz.

The transmission properties of the unit cell are simulated by using the commercial time-domain package CST Microwave Studio, in which the boundaries are set to be periodic along the *x* and *y* directions. To easily modulate the phase, the inner radius *r* is varied and the outer radius is set to *R* = 5.8 mm.  Figure 2(a) depicts the simulated transmission intensity and phase difference for *r* = 4.58 mm. The red dot line represents 0.7 transmission coefficient. It can be clearly observed that the phase difference can be over 360 degrees, while the transmission coefficient is over 0.7. This characteristic makes the unit cell structure promising in metasurface design.

Using simulations, a transmission coefficient of unit cells greater than 0.7 is acquired at 10 GHz for an inner radius change from 1.5 to 4.7 mm. Thus, the operation frequency of the metasurface is set to 10 GHz. Referring to the OAM beam generation method using an antenna array, the metasurface arranged with unit cells in key positions can engender the OAM beam. A circular arrangement is usually used in the antenna array for the OAM beam generation. Herein, the same distribution mode is adopted. In this distribution mode, more unit cells engender a larger size. However, a sufficient number is essential for the OAM beam generation. Considering the size of each unit cell and the entire metasurface, eight unit cells are suitable for the smallest distribution circle with a radius of 18 mm. To generate the OAM beam, the number of antennas *N* and phase difference *∆φ* need to satisfy the following condition [38]:

(1) $$\begin{array}{c} \Delta \varphi =l\cdot \frac{2\pi }{N}, \end{array}$$

where *l* is the mode of the OAM beam.

In this study, *N* = 8 and the phase difference is determined as *∆φ* = *l* · *π*/4 for the design of the metasurface. Therefore, eight kinds of unit cells with a 45°-phase difference are required. According to the above simulations, these unit cells are easily found by changing the inner radius *r* at the frequency of 10 GHz. As shown in Figure 2(b), the phase of the transmitted EM wave increases in steps of 45° for the *r* set to 4.78 mm, 4.69 mm, 4.58 mm, 4.40 mm, 4.10 mm, 3.57 mm, 2.52 mm, and 1.62 mm in turns.

**Figure 3:**
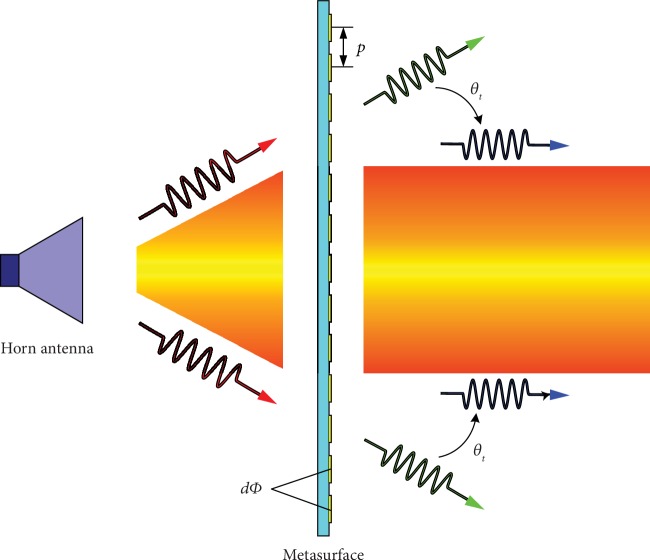
Schematic illustration of decreasing the divergence angle.

A horn antenna is often used as an exciting source of metasurfaces. Nevertheless, the EM wave emitted from a horn antenna presents a certain divergence angle. This has to be considered during the design of the metasurface to avoid repercussions on the transmitted EM wave. As shown in Figure 3, the red arrows represent the EM wave emitted from the horn antenna and the green arrows represent the transmitted EM wave. Both of them have the same divergence angle.

Phase gradient metasurfaces can cause the steering of EM waves. In our design, the divergence angle issue can be improved by adding the phase gradient in the radial directions, using the generalized Snell's law of refraction [39, 40]:

(2) $$\begin{array}{c} \sin\left(\theta_{t}\right)n_{t}-\sin\left(\theta_{i}\right)n_{i}=\frac{\lambda_{0}}{2\pi }\frac{d\Phi }{dx}, \end{array}$$

where *θ*_*t*_ and *θ*_*i*_ are the angles of refraction and incidence, respectively, *n*_*t*_ and *n*_*i*_ are the refractive indices of the two media outside the metasurface, *λ*_0_ is the vacuum wavelength, and *dΦ*/*dx* is the constant gradient of the phase. In free space, *n*_*t*_ = *n*_*i*_ = 1, *dΦ*/*dx* = *dΦ*/*p*, and the desired divergence angle engenders *θ*_*t*_ = 0; thus, the phase difference can be described as follows:

(3) $$\begin{array}{c} d\Phi =-\sin\left(\theta_{i}\right)\cdot 2\pi \cdot \frac{p}{\lambda_{0}}. \end{array}$$

**Figure 4:**
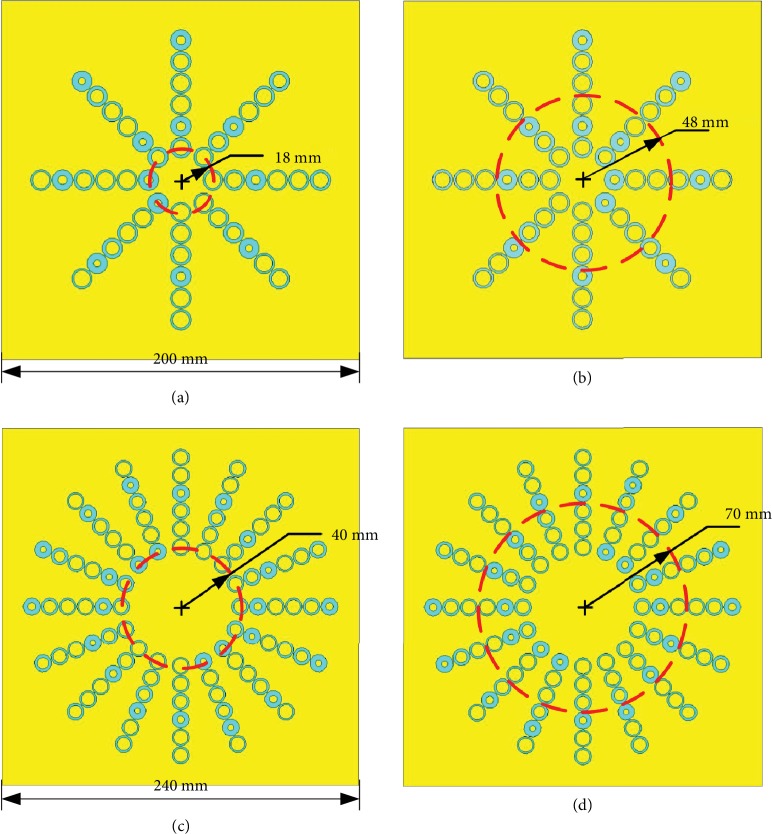
Structure models of the metasurface antennas with different modes: (a) *l* = 1, (b) *l* = −1, (c) *l* = 2, and (d) *l* = −2.

The detailed value of *dΦ* can be calculated if the setup of the experiment (incident angle *θ*_*i*_) is known. According to the above discussion, the variation of the parameter *r* can also cause the phase difference. Moreover, the phase gradient is distributed in the radial directions, while the phase difference of the OAM beam generation is required in the circle direction. These two kinds of phase variations are independent and can be satisfied simultaneously. To satisfy these two kinds of phase variations, the simulation models of the metasurface antennas are built and shown in Figure 4.

The side lengths of the entire metasurface antennas with *l* = ±1 and ±2 are 200 mm and 240 mm, respectively. The radius of the first distribution circle also increases from 18 mm to 40 mm due to the doubling of the unit cell number. In our design, there are six unit cells in each radial direction to ensure the effectiveness of the small divergence angle (Figures [Supplementary-material supplementary-material-1] and [Supplementary-material supplementary-material-1] in the Supporting Information). The details of the design will be illustrated by the example of the metasurface antenna with *l* = +1. In this case, the distance between the excitation source and the metasurface antenna is 60 mm. The centers of the unit cells in each radial direction are arranged in a circle with a radius of 48 mm. Therefore, the incident angle *θ*_*i*_ is 38.66°. The period of the unit cell *p* is 12 mm, the vacuum wavelength *λ*_0_ is 30 mm (*f* = 10 GHz). Therefore, the value of *dΦ* is approximately equal to −*π*/2. Besides, the phase difference *∆φ* is equal to *π*/4. The parameters of every unit cell can be determined according to the simulated results shown in Figure 2(b).

**Figure 5:**
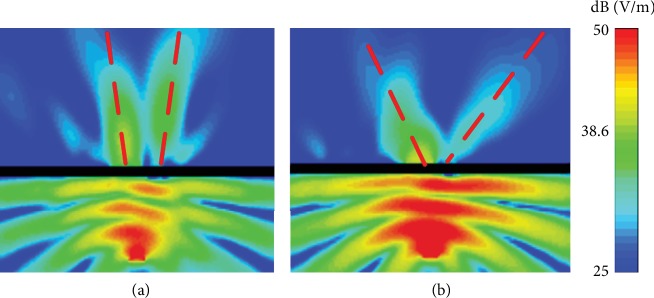
Simulated electric field (*E*_*x*_) amplitude distribution (a) with and (b) without phase gradient in the radial directions.

To verify the effect of the small divergence angle, the proposed metasurface is compared with a metasurface which does not present a phase gradient in the radial directions. The other parameters are the same as the parameters used in the above description. Both metasurfaces can generate an OAM beam with *l* = +1 at 10 GHz. The simulated electric field amplitude distributions in the *xoz* plane are shown in Figure 5. The black strip in the middle of the figure represents the metasurface. The lower part of the picture represents the excitation signal which shows a certain divergence angle. The four red dashed lines indicate the divergence angles. It can be clearly observed that the divergence angle of the proposed metasurface antenna is smaller than that of the other antenna. In fact, the phase gradient plays a role in decreasing the divergence angle. Specifically, the divergence angles in Figures 5(a) and 5(b) are about 9 degrees and 37 degrees, respectively. In the calculations of the phase gradient, the refraction angle *θ*_*t*_ is set as 0 to implement the desired divergence angle (*θ*_*t*_ = 0). The non-zero divergence angle originates from the natural characteristic of OAM beams.

## 3. Results and Discussion

Numerical predictions of the phase distributions and radiation patterns for the proposed metasurface were simulated.The parameters and operation frequency are the same as the ones indicated in the description in Section 2. For the mode *l* = ±1, the phase observation plane with an area of 200 mm × 200 mm is set 300 mm away from the metasurface due to the limitation of the computer resources. For the mode *l* = ±2, the observation plane with an area of 600 mm × 600 mm is set 600 mm away from the metasurface.

**Figure 6:**
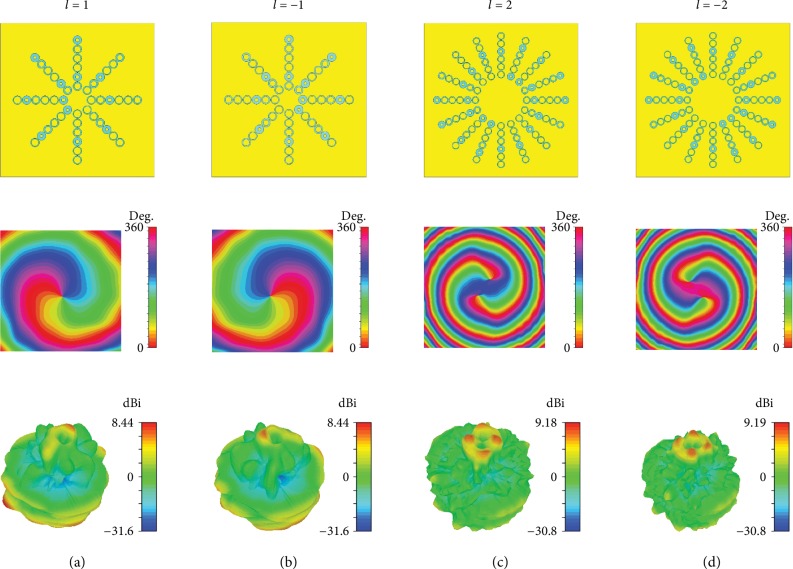
Configurations, phase distributions (*xoy* plane), and radiation patterns with modes of (a) *l* = 1, (b) *l* = −1, (c) *l* = 2, and (d) *l* = −2.

**Figure 7:**
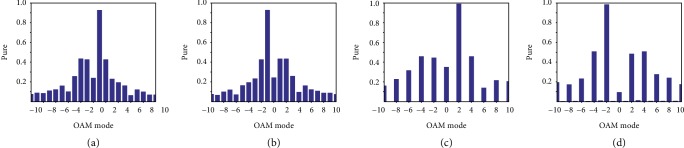
Histograms of the OAM spectrum for the OAM mode: (a) *l* = 1, (b) *l* = −1, (c) *l* = 2, and (d) *l* = −2.

The simulated results are shown in Figure 6. The figures in the first row represent the configurations of the metasurfaces with modes of ±1 and ±2. Here, the same phase difference *∆φ* is applied for the generation of OAM beams with modes of ±1 and ±2. Thus, the number of unit cells is doubled for the modes of ±2. For convenience, all the phase gradients *dΦ*/*p* are set to the same value of (−*π*/2)/12. To achieve the small divergence angle, the positions of the exciting source should be modified to obtain the same incidence angle. The second and third rows of Figure 6 present the phase distributions and radiation patterns of the generated OAM beams, respectively. The phase distributions exhibit a vortex shape, and the radiation patterns show a deep pit surrounded by a high-intensity ring, which satisfies the OAM characteristics of *l* = ±1 and ±2. In addition, the OAM spectrums with different modes are shown in Figure 7. The purities of the desired OAM beams are over 0.8, which illustrates the quality of the generated OAM beams.

**Figure 8:**
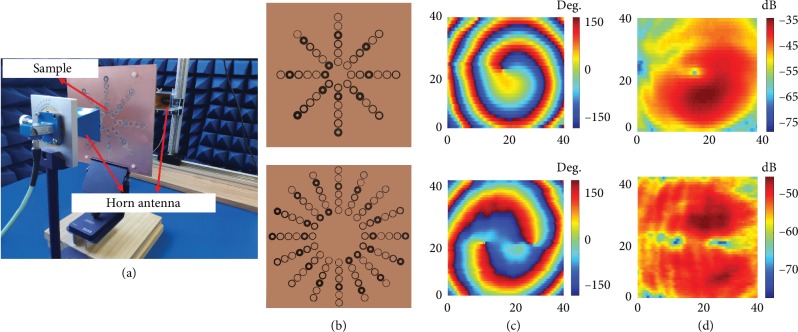
(a) Setup of the OAM beam measurement, (b) complete fabricated structure of the metasurface antennas with *l* = −1 and 2, (c) measured near-field phase distributions of the OAM beams, and (d) measured near-field intensity distributions of the OAM beams.

To verify the above analysis and calculation, an OAM beam measurement system is designed, and the setup is depicted in Figure 8(a). Here, a horn antenna connected to the port 1 of the vector network analyzer (VNA) is used as the exciting source. As shown in Figure 8(b), two prototypes of the metasurface antennas with the modes of -1 and 2 are fabricated and placed in front of the exciting source with the appropriate distances. Another horn antenna is connected to the port 2 of the VNA and is used as a receiving antenna to measure the near-field phase and intensity distributions of the generated OAM beams. The measured signals are then postprocessed by the VNA. The operation frequency is 10 GHz. Figures 8(c) and 8(d) represent the measured phase and intensity distributions. These measured results correspond to the simulated results presented in Figure 6. However, a small deformation is noted in the measured results due to errors of machining and experimental operations. The major features, such as spiral-shaped phase distributions and doughnut-shaped intensity distributions, can be clearly observed. Therefore, the capability of the proposed metasurface antenna to generate OAM beams is clearly demonstrated by this experiment.

## 4. Conclusion

In summary, we designed a simple metasurface antenna presenting the capacity in generating small-divergency-angle OAM beams. Herein, the circular arrangement of the unit cells was adopted in the design of the metasurface. Compared with the conventional metasurfaces, the circular arrangement seems more compact in the OAM beam generation, which greatly simplifies the structure of the traditional metasurface. The phase gradient in the radial directions was proposed to decrease the divergence angle of the generated OAM beams. It successfully allows achieving both the OAM beam generation and the small divergence angle effect. According to the generalized Snell's law of refraction, a series of rigorous derivations and calculations were performed. The small divergence angle and OAM beam generation were verified by simulations. The simulated electric field of the metasurface antenna with a phase gradient in the radial directions presents a small divergence angle. Moreover, two prototypes of the proposed metasurface antenna were fabricated, and an experimental platform was designed to validate the effectiveness of the generation of OAM beams. The simulated and measured results were in accordance and demonstrate the validity of the design.

## Supplementary Materials

Supplementary Materials Fig. S1: (a) configurations, (b) phase distributions (*xoy* plane), and (c) radiation patterns with different numbers of unit cells in the radial direction. Fig. S2: (a) prototypes of the metasurface antennas, (b) measured phase distributions (*xoy* plane), and (c) measured near-field amplitude distributions with different numbers of unit cells in the radial direction.
