# Supplementary material for: A Small-Divergence-Angle Orbital Angular Momentum Metasurface Antenna
Source: Research (Wash D C). 2019 Nov 15;2019:9686213. doi: 10.34133/2019/9686213 (PMC6946278; doi:10.34133/2019/9686213)
Supplement: Supplementary Materials — Fig. S1: (a) configurations, (b) phase distributions (xoy plane), and (c) radiation patterns with different numbers of unit cells in the radial direction. Fig. S2: (a) prototypes of the metasurface antennas, (b) measured phase distributions (xoy plane), and (c) measured near-field amplitude distributions with different numbers of unit cells in the radial direction. [file 9686213.f1.pdf]

Research
OFFICIAL JOURNAL OF CAST

# Manuscript Template

## FRONT MATTER

## Title

**Long: A Small-divergence-angle Orbital Angular Momentum Metasurface Antenna**

**Short: Orbital Angular Momentum Metasurface Antenna**

## Authors

Jianchun Xu[1], Ke Bi[1,4,*], Ru Zhang[1,5], Yanan Hao[1], Chuwen Lan[1], Klaus D. McDonald-Maier[2], Xiaojun Zhai[2], Zidong Zhang[3,*], and Shanguo Huang[1,*]

## Affiliations

[1] State Key Laboratory of Information Photonics and Optical Communications, School of Science, Beijing University of Posts and Telecommunications, Beijing 100876, China

[2] School of Computer Science and Electronic Engineering, University of Essex, Colchester CO4 3SQ, United Kingdom

[3] Key Laboratory for Liquid-Solid Structural Evolution and Processing of Materials (Ministry of Education), Shandong University, Jinan 250061, China

[4] Beijing University of Posts and Telecommunications Research Institute, Shenzhen 518057, China

[5] Beijing Key Laboratory of Space-ground Interconnection and Convergence, Beijing University of Posts and Telecommunications, Beijing 100876, China

*Correspondence should be addressed to: Ke Bi; bike@bupt.edu.cn, Zidong Zhang; zhangzidong@sdu.edu.cn, Shanguo Huang; shghuang@bupt.edu.cn

## Simulations

The unit cell number is one of the most important parameters in the metasurface antenna design. A small number represents a simple structure, while a large number is favorable to achieve small divergence angles and standard vortex-shaped front waves. To determine the appropriate unit cell number, several simulations of metasurface antenna designs with various unit cell numbers in the radial direction are obtained using CST Microwave Studio. In the simulations, the unit cell number changes from 1 to 5. The configurations of the designs and the simulated results are shown in **Fig. S1**. The phase distributions and radiation patterns of all the designs demonstrate obvious features of the orbital angular momentum beam, even the unit cell number is 1. From the **Figures S1(b)** and **S1(c)**, it can clearly be observed that when the number increases, the vortex shape of the phase distribution becomes standard, and the divergence angle is significantly improved. The phase modification of the unit cell in the radial direction can be greater than 2π when the number is greater than 4. Therefore, the improvement effect becomes less evident as the number is greater than 4. The 2π phase modification represents the necessary condition to obtain the small divergence angle. In order to ensure the small divergence angle, six unit cells were created in each radial direction of our design.

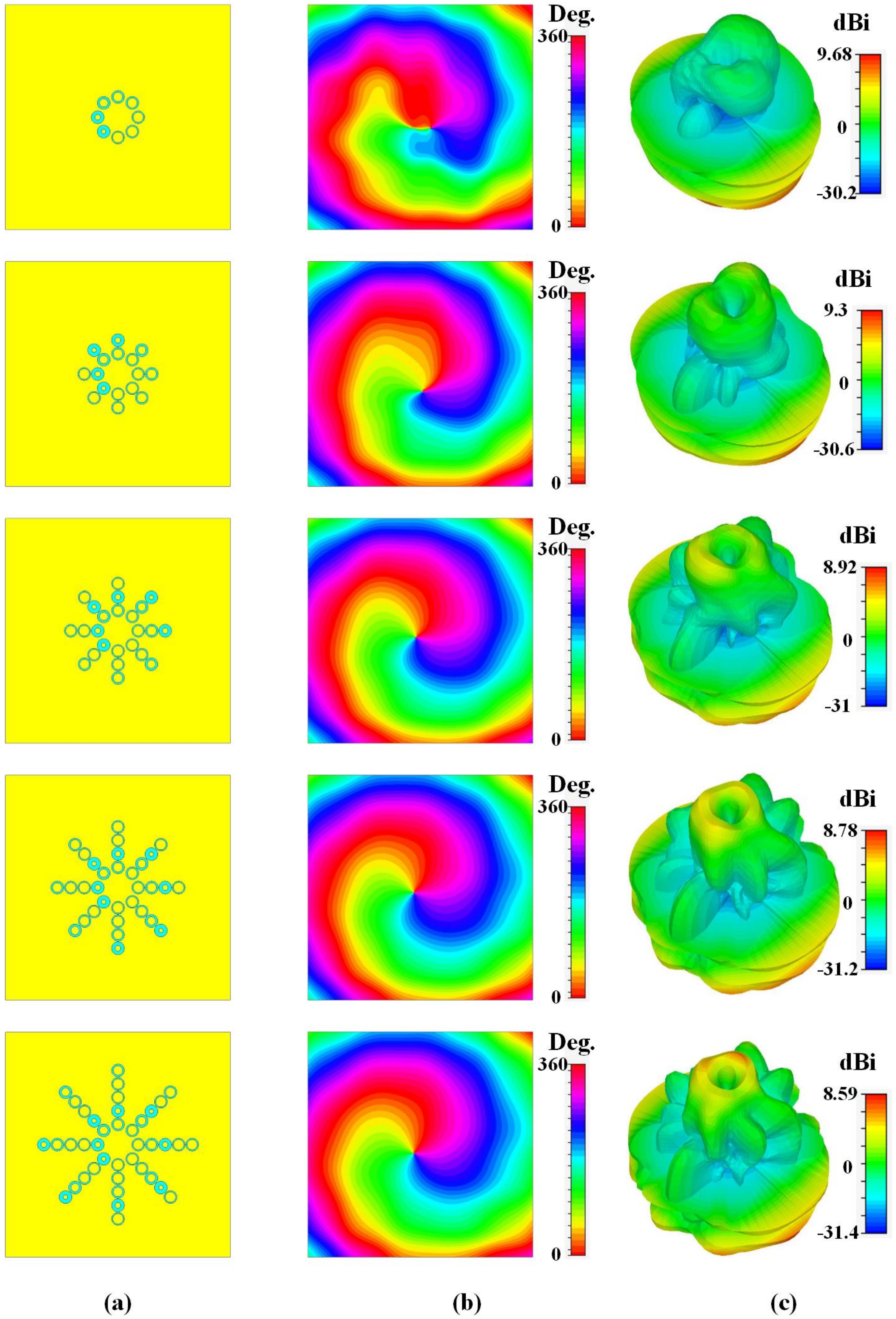


**Fig. S1** (a) Configurations, (b) phase distributions (*xoy* plane), and (c) radiation patterns with different numbers of unit cells in the radial direction.

## Measurements

To verify the above simulations, the metasurface antennas with different unit cells numbers of *n* = 1, 3, and 5 are fabricated and their near-field phase and amplitude distributions are measured. The prototypes of the metasurface antennas and the measured results are shown in **Fig. S2**. For clear visions, the observation planes with an area of 500 mm × 500 mm are set 300 mm away from the metasurface antennas. And the operating frequencies are also 10 GHz. The obvious vortex shaped phase distributions and doughnut shaped amplitude field distribution showed in **Figures S2(b)** and **S2(c)** are the remarkable characteristics of the OAM beams, which demonstrate the generations of the OAM beams with *l* = 1. It is worthy to note that the amplitude distribution becomes more centralized as the unit cells number increases. Moreover, the received amplitude is enhanced due to the concentration. Therefore, the sufficient unit cells number is the guarantee of the small divergence angle and high transmissivity.

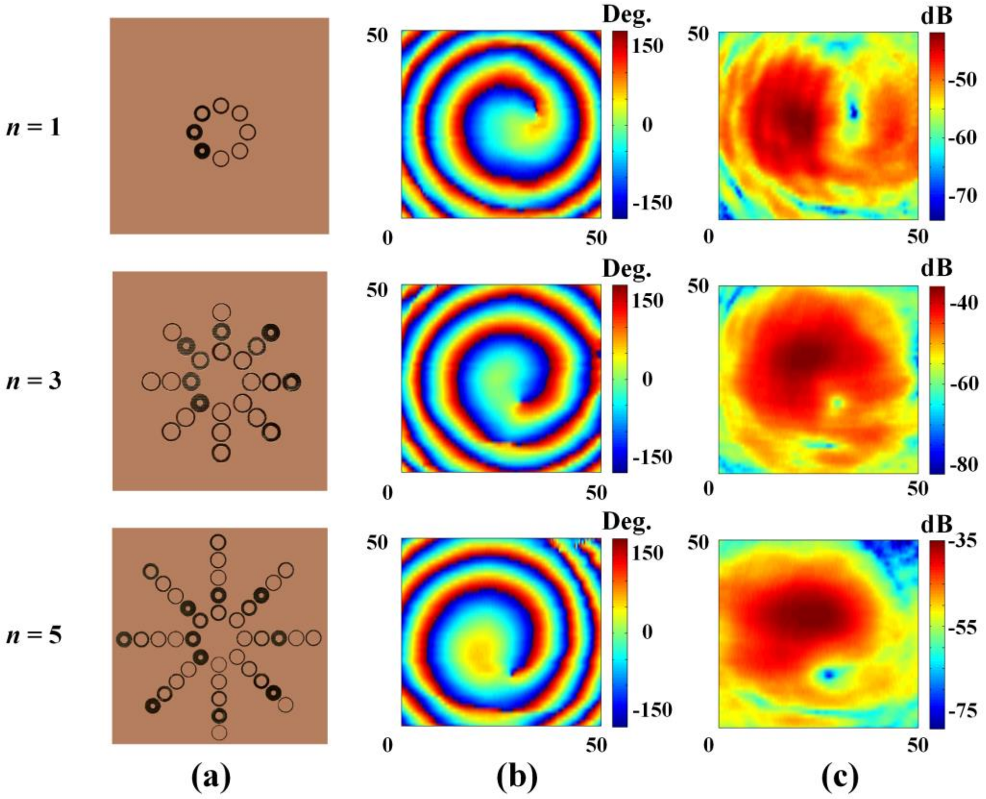

**Fig. S2** (a) Prototypes of the metasurface antennas, (b) measured phase distributions (*xoy* plane), and (c) measured near-field amplitude distributions with different numbers of unit cells in the radial direction.
